# Speech and gait abnormalities in motor subtypes of de‐novo Parkinson's disease

**DOI:** 10.1111/cns.14158

**Published:** 2023-03-21

**Authors:** Jan Rusz, Radim Krupička, Slávka Vítečková, Tereza Tykalová, Michal Novotný, Jan Novák, Petr Dušek, Evžen Růžička

**Affiliations:** ^1^ Department of Circuit Theory, Faculty of Electrical Engineering Czech Technical University in Prague Prague Czechia; ^2^ Department of Neurology and Centre of Clinical Neuroscience, First Faculty of Medicine Charles University and General University Hospital Prague Czechia; ^3^ Department of Neurology & ARTORG Center Inselspital, Bern University Hospital University of Bern Bern Switzerland; ^4^ Faculty of Biomedical Engineering Czech Technical University in Prague Prague Czechia; ^5^ Department of Anthropology and Human Genetics, Faculty of Science Charles University Prague Czechia; ^6^ Department of Radiology, First Faculty of Medicine Charles University and General University Hospital Prague Czechia

**Keywords:** dysarthria, gait, Parkinson's disease, postural instability gait difficulty, speech disorder

## Abstract

**Aim:**

To investigate the presence and relationship of temporal speech and gait parameters in patients with postural instability/gait disorder (PIGD) and tremor‐dominant (TD) motor subtypes of Parkinson's disease (PD).

**Methods:**

Speech samples and instrumented walkway system assessments were acquired from a total of 60 de‐novo PD patients (40 in TD and 20 in PIGD subtype) and 40 matched healthy controls. Objective acoustic vocal assessment of seven distinct speech timing dimensions was related to instrumental gait measures including velocity, cadence, and stride length.

**Results:**

Compared to controls, PIGD subtype showed greater consonant timing abnormalities by prolonged voice onset time (VOT) while also shorter stride length during both normal walking and dual task, while decreased velocity and cadence only during dual task. Speaking rate was faster in PIGD than TD subtype. In PIGD subtype, prolonged VOT correlated with slower gait velocity (*r* = −0.56, *p* = 0.01) and shorter stride length (*r* = −0.59, *p* = 0.008) during normal walking, whereas relationships were also found between decreased cadence in dual task and irregular alternating motion rates (*r* = −0.48, *p* = 0.04) and prolonged pauses (*r* = −0.50, *p* = 0.03). No correlation between speech and gait was detected in TD subtype.

**Conclusion:**

Our findings suggest that speech and gait rhythm disorder share similar underlying pathomechanisms specific for PIGD subtype.

## Graphical abstract

PIGD subtype manifested more severe temporal speech and gait impairment compared to the TD subtype. Strongest relationships were found between prolonged voice onset time and slower gait velocity and shorter stride length. Our findings suggest that speech and gait rhythm disorder share similar underlying pathomechanisms specific for PIGD subtype.

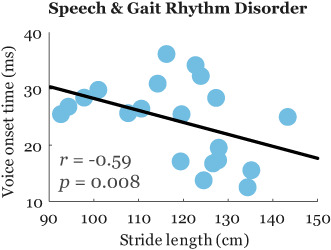

## 1 INTRODUCTION

Parkinson's disease (PD) is a progressive neurodegenerative synucleinopathy that is manifested with a diverse spectrum of motor and nonmotor symptoms.^1^ Speech and gait are automatic motor activities that are frequently affected by PD, occurring in early and even prodromal disease stages.^2^, ^3^ Among dysphonia, hypokinetic articulation, and dysprosody, hypokinetic dysarthria in PD is manifested by various speech timing abnormalities, including rhythm deficiency, prolonged pauses, altered speech rate, and others.^4^, ^5^ Gait impairment is a debilitating feature of PD mainly characterized by a slower velocity with shorter stride length and a compensatory increase in walking cadence.^6^

These findings imply that speech timing abnormalities may have some points in common with spatiotemporal gait disturbances. Speech and walking velocity might decrease in some PD patients, while inappropriate pauses and impaired cadence might be the result of a similar rhythmic disorder. It is thus debated to what extent speech and gait abnormalities in PD are the result of the same pathophysiology. Yet, objective data documenting relationships between speech and gait abnormalities in PD are scarce.^7^, ^8^, ^9^, ^10^, ^11^ Most of the available studies have found correlations between speech dysfunction and freezing of gait in advanced PD patients on dopaminergic medication.^7^, ^8^, ^9^ In addition, a correlation between rater‐based gait festination and syllable repetition task‐based oral festination has been observed.^10^ The association between natural speech and walking characteristics such as velocity and rhythmicity has only been reported in PD patients treated with subthalamic nucleus deep brain stimulation.^11^ However, pharmacological and surgical interventions as well as the various pace of disease progression might pose different influences on speech and gait patterns.^12^, ^13^ Potential speech and gait relations in de‐novo, untreated PD patients have not yet been explored.

One possible reason for the ambiguities about the existence of similar brain processes underlying speech and gait motor functions might be the unequal proportion and severity of speech and gait disorder across individual PD patients.^6^, ^13^ These interindividual disparities in speech and gait patterns are likely related to various clinical subtypes of PD with differences in disease profiles and progression rates.^14^, ^15^ Such heterogeneity seems to be consistent with the existence of motor subtypes, most commonly divided into the postural instability/gait disorder (PIGD) or tremor dominant (TD) subtypes.^15^ By definition, patients with the PIGD subtype exhibit more balance and gait impairment than patients with the TD subtype. Nevertheless, more significant speech impairment in the PIGD compared to the TD subtype has also been reported,^16^ indicating that certain connections exist between speech and gait disorders in PD.

Therefore, we aimed to compare temporal speech and gait parameters between the PIGD and TD motor phenotypes of de‐novo PD patients and healthy controls. Additionally, we aimed to examine potential correlations between the temporal speech and gait parameters in both PD subtypes. We hypothesized that both speech and gait would be affected predominantly in the PIGD subtype and that timing abnormalities of speech and gait would be interrelated.

## 2 METHODS

### 2.1 Study design

From 2016 to 2022, a consecutive group of de‐novo, drug‐naive PD patients was recruited. PD patients were diagnosed based on the Movement Disorder Society clinical diagnostic criteria for PD^17^ and investigated before the introduction of dopaminergic therapy. This study is part of a longitudinal project “biomarkers in PD (BIO‐PD)” aimed to collect a large representative sample of de‐novo PD patients; the detailed protocol of this project has been described previously.^18^ The inclusion criteria for PD patients were as follows: (i) fulfilling criteria to allow inclusion into the TD or PIGD group, (ii) native Czech language speaker, (iii) no history of therapy with antiparkinsonian medication, (iv) no history of communication or significant neurological disorders unrelated to PD, and (v) no current or past involvement in any speech therapy. The exclusion criteria were as follows: (i) clinical or imaging signs of atypical parkinsonism and (ii) normal finding on dopamine transporter single‐photon emission computed tomography examination. The healthy control subjects were recruited from the general community through advertisements. To be eligible for the study, controls had to be free of speech disorder, motor neurologic disorder, active oncologic illness, and abuse of psychoactive substances. The clinical evaluation of each subject included (i) structured clinical interview focused on personal and medical history, history of drug and substance intake, and current drug usage, (ii) quantitative testing of PD severity using the Movement Disorder Society‐Unified Parkinson Disease Rating Scale (MDS‐UPDRS) part II and III,^19^ and (iii) cognitive testing with the Montreal Cognitive Assessment (MoCA).^20^ All evaluations of clinical scales were performed by a neurologist experienced in movement disorders (P.D.). Symptom duration was estimated based on the self‐reported occurrence of the first motor symptoms.

### 2.2 PD Motor subtypes classification

The PD patients were categorized into PIGD or TD clinical subtypes according to the method proposed by Stebbins et al.^21^ in which MDS‐UPDRS items for TD and PIGD designations are used to calculate the mean MDS‐UPDRS TD and MDS‐UPDRS PIGD scores. Specifically, the ratio of the mean MDS‐UPDRS tremor score (i.e., mean from the following 11 MDS‐UPDRS items: 2.10 [tremor] and 3.15.‐3.18 [all items related to postural, kinetic, and rest tremor]) to the mean MDS‐UPDRS PIGD score (i.e., mean from the following 5 MDS‐UPDRS items: 2.12 [walking and balance], 2.13 [freezing], 3.10 [gait], 3.11 [freezing of gait], and 3.12 [postural stability]) were used to define TD patients (ratio ≥1.15) or PIGD patients (ratio ≤0.9). In addition, patients who had a nonzero value in the numerator and a zero in the denominator were classified as TD, while patients with a zero in the numerator and a nonzero value in the denominator were classified as PIGD.

### 2.3 Speech assessment

Speech recordings were performed in a quiet room with a low ambient noise level using a head‐mounted condenser microphone (Beyerdynamic Opus 55, Heilbronn, Germany) placed approximately 5 cm from the subject's mouth. Speech signals were sampled at 48 kHz with 16‐bit resolution. Each subject was recorded during a single session with a speech specialist. Participants were instructed to perform three vocal tasks including (i) repetition of the syllable/pa/at least 20 times at a comfortable, self‐determined, steady pace without acceleration or deceleration, (ii) fast/ta/syllable repetition for approximately 5 s per one breath, and (iii) reading a short paragraph of standardized text composed of 80 words. These vocal tasks provide comprehensive information necessary for the objective description and interpretation of motor speech disorders.^22^ Each task was repeated twice per participant.

### 2.4 Gait assessment

All subjects completed an extended Timed Up & Go Test (TUG): to rise from a chair, walk 10 meters at the usual preferred walking speed, turn, walk back, and sit down again. TUG was performed twice. A 5.15 m long and 0.9 m wide pressure walkway (Platinum model GAITRite®, CIR System Inc.) was placed 2.43 m from the chair in the middle of the straight gait walkway. Steady gait was captured twice in each TUG measurement: before the turn and after the turn. Pre‐turn and post‐turn gait were acquired as separate recordings on the walkway. The participants were instructed to walk under two different conditions: (i) at a normal pace (hereafter, single‐task), and (ii) at a normal pace while counting down from 100 by sevens (hereafter, dual task). The rationale for including dual‐task gait measure in relation to single‐task speech assessment is that dual‐task represents a routine way of testing gait in PD. In particular, many gait alterations in early‐stage PD become apparent or exaggerated when patients are asked to walk and do another task at the same time.^6^

### 2.5 Acoustic speech and instrumental gait features

We performed a quantitative acoustic vocal assessment of seven distinct temporal speech dimensions (Table 1). Acoustic analysis was preferred because it provides objective, sensitive, and quantifiable information for the precise assessment of speech performance from the very early stages of PD.^2^ Considering rhythm in syllable repetition, we examined *pace acceleration* using rhythm acceleration (RA) and *pace instability* using rhythm instability (RI). For the fast syllable repetition paradigm, we extracted *slow alternating motion rates* (AMR) using the diadochokinetic rate (DDKR), *irregular AMR* using diadochokinetic irregularity (DDKI), and *imprecise consonants* using voice onset time (VOT). Using reading passages, we calculated *prolonged pauses* using the duration of pause intervals (DPI) and *abnormal articulation rate* using net speech rate (NSR). The final speech values used for the statistical analyses were averaged across two repetitions to provide greater speech assessment stability.^22^ Comprehensive algorithmic details on individual acoustics measures have been reported previously.^23^ Also, the accuracy of algorithms for the identification of temporal intervals has been thoroughly tested in previous studies.^5^, ^23^, ^24^

**TABLE 1:** Definition of outcome measures.

Altered dimension	Objectivefeature	Definition	Pathophysiological interpretation with respect to hypokinesia
Speech [vocal task]			
Pace acceleration [rhythmic syllable repetition]	RA	Rhythm acceleration, defined as the as the gradient of the regression line obtained through regression performed on gaps duration between two consecutive syllables, with values greater than 0 indicating accelerated rhythm. Performance (ms/s).	Hypokinesia leads to oral festination
Pace instability [rhythmic syllable repetition]	RI	Rhythm instability, defined as the sum of absolute deviations of gap duration between two consecutive syllables from the regression line, weighted to the total speech time (%)	Hypokinesia causes deficits in speech timing
Slow alternating motion rates [fast syllable repetition]	DDKR	Diadochokinetic rate, defined as the number of syllable vocalizations per second (syll/s)	Hypokinesia of speech apparatus makes the movements of articulators slower
Irregular alternating motion rates [fast syllable repetition]	DDKI	Diadochokinetic irregularity, defined as the standard deviation of distances between following syllable nuclei (1/s)	Hypokinesia causes deficits in speech timing
Imprecise consonants [fast syllable repetition]	VOT	Voice onset time, defined as the length of the entire consonant from initial burst to vowel onset (ms)	Hypokinesia causes slowing of lip and tongue movements, leading to a longer time required to pronounce individual consonants
Prolonged pauses [reading passage]	DPI	Duration of pause intervals, defined as the median length of pause intervals (ms)	Hypokinesia of speech apparatus makes initiating of speech difficult, leading to prolonged pause intervals
Abnormal articulation rate [reading passage]	NSR	Net speech rate, defined as the total number of syllables divided by the total duration of speech after removal of pauses (syll/s)	Impaired control of orofacial muscles leads to a decrease in speech rate
Gait			
Slow velocity	Gait velocity	Gait velocity, defined as the distance walked per second (cm/s)	Hypokinesia leads to slower walking speed
Increased cadence	Cadence	Cadence, defined as the number of steps per minute (steps/min)	Hypokinesia leads to compensatory increase in walking cadence
Shorter stride length	Stride length	Stride length, defined as the distance from a point of the ground contact of one foot to the point of the ground contact of the same foot (cm)	Hypokinesia causes shorter stride length

Furthermore, we selected three representative gait measures provided by GAITRite including gait velocity, cadence, and stride length (Table 1).^6^ *Gait velocity* was defined as a distance measured between the first footfall and the last footfall divided by the time elapsed between them, *cadence* as a number of steps taken within a minute provided, and str*ide length* as the distance between the two consecutive footprints of the same foot. Gait parameters for each record (i.e., walk forward and walk back across 2 trials) were averaged.

### 2.6 Statistical analysis

An ad hoc power analysis with one covariate (group) given a large effect size (Cohen's *f* of 0.4) with the error probability *α* set at 0.05 and a false negative rate *β* set at 0.2 (i.e., power of 0.8) indicated a recommended minimum overall sample size of 66 for three groups.^25^ The Shapiro–Wilk test was used to evaluate the normality of distributions. Kruskal–Wallis test with the post‐hoc Fisher least significant difference test was preferred to assess group differences due to presence of outliers across certain measures. Since there was wide variability in age among PD patients, Spearman's partial correlation analysis controlled for age was performed to test for significant relationships between the speech and gait variables across PD subtypes. The level of significance was set to *p* < 0.05. All statistical analyses were performed in MATLAB (MathWorks).

## 3 RESULTS

### 3.1 Clinical and perceptual speech description

The TD group consisted of 40 patients (28 men) with a mean age of 63.2 (SD 10.1, range 39–80) years, PIGD group of 20 patients (13 men) with a mean age of 63.9 (SD 12.4, range 36–80) years, and control group of 40 subjects (28 men) with a mean age of 62.5 (SD 7.7, range 45–81) years sex‐ and age‐matched to both PD subtypes (Table 2). TD and PIGD groups had similar symptom duration, motor severity, and cognitive function. MDS‐UPDRS III showed similar severity between TD and PIGD on perceptual speech disorder and freezing of gait items while gait disorder item showed higher mean values in the PIGD group (*p* = 0.006).

**TABLE 2:** Clinical characteristics of participants.

	TD	PIGD	Controls	*p*‐value
General				
Male sex	28/40 (70%)	13/20 (65%)	28/40 (70%)	0.91
Age (years)	63.2 (10.1, 39–80)	63.9 (12.4, 36–80)	62.5 (7.7, 45–81)	0.44
Symptom duration (years)	1.7 (1.2, 0.4–5.3)	1.6 (1.3, 0.1–5.2)	n/a	0.73
MoCA	24.4 (2.9, 17–30)	25.3 (3.1, 17–28)	25.3 (2.2, 19–29)	0.23
Motor symptoms				
MDS‐UPDRS II	7.1 (4.5, 1–16)	8.9 (4.7, 3–20)	0.6 (1.1, 0–5)	<0.001
MDS‐UPDRS III	29.8 (14.2, 10–70)	30.4 (12.1, 10–52)	3.1 (2.8, 0–13)	<0.001
Slight speech disorder^a^	21/40 (53%)	14/20 (70%)	0/40 (0%)	<0.001
Slight gait disorder^a^	13/40 (33%)	14/20 (70%)	1/40 (3%)	<0.001^b^
Slight freezing of gait^a^	2/40 (5%)	3/20 (15%)	0/40 (0%)	0.04

*Note*: Data are mean (SD, range) including *p*‐values analyzed using Kruskal–Wallis test or number/sample size (%) Including *p*‐values analyzed using chi‐squared test.

Abbreviations: MDS‐UPDRS, Movement Disorder Society Unified Parkinson's Disease Rating Scale; MoCA, Montreal Cognitive Assessment; n/a, not applicable; PIGD, postural instability/gait difficulty subtype of Parkinson's disease; TD, tremor dominant subtype of Parkinson's disease.

^a^ The respective item of MDS‐UPDRS III was equal to 1 (i.e., slight disorder); remaining subjects were scored by 0 points (i.e., without disorder).

^b^ Significantly different between TD and PIGD group with *p* < 0.01.

The primary features of dysarthria in both PD subtypes that were perceptibly included mainly monopitch and monoloudness. The PIGD group manifested more frequently worsened quality of voice and imprecise articulation than the TD group. No PD patient presented with palilalia and freezing of speech.

### 3.2 Group differences

**FIGURE 1:**
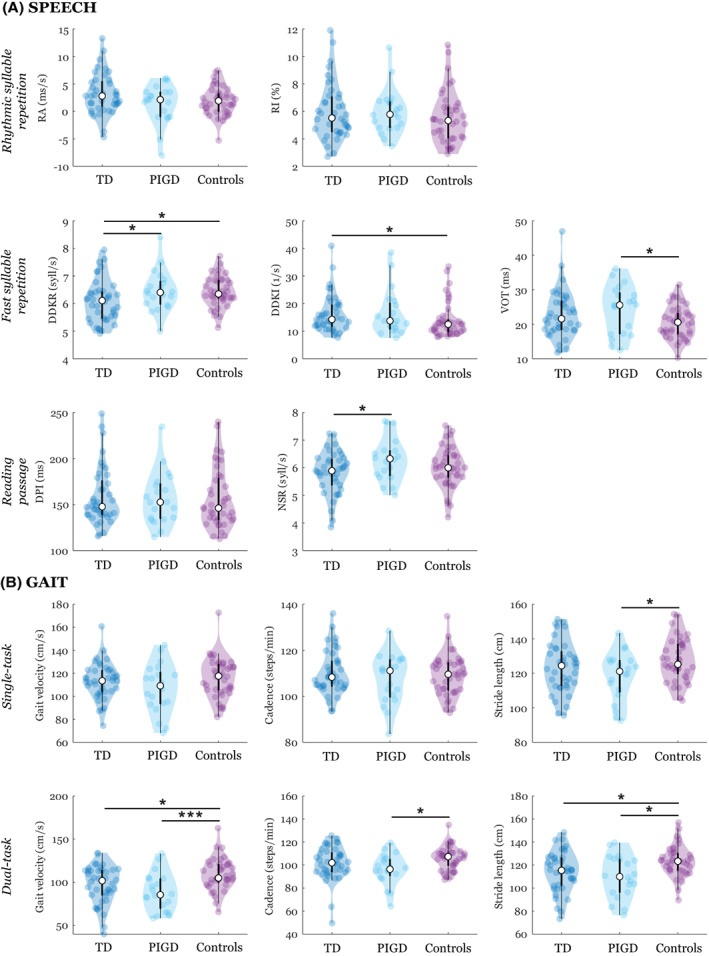
Violin plots of individual speech and gait dimensions. The plot shows the median (indicated by the open circle), the first through the third interquartile range (the thick, solid vertical band), estimator of the density (color vertical curves) of the individual scores in each group (comparable to a box plot, except that the distribution of the variable is illustrated as density curves) and individual scores (color‐filled circles). Statistically significant differences between groups: *p < 0.05, **p < 0.01, ***p < 0.001. DDKI, diadochokinetic irregularity; DDKR, diadochokinetic rate; DPI, duration of pause intervals; NSR, net speech rate; PIGD, postural instability/gait difficulty subtype of Parkinson's disease; RA, rhythm acceleration; RI, rhythm instability; TD, tremor dominant subtype of Parkinson's disease; VOT, voice onset time.

In the speech assessment, patients with the TD subtype showed slower AMR (DDKR: *p* = 0.04) as well as slower articulation rate (NSR: *p* = 0.03) compared to the PIGD subtype (Figure 1A; Video S1). AMR in the TD subtype was also significantly slower (DDKR: *p* = 0.04) and irregular (DDKI: *p* = 0.03) than in controls. PIGD subtype showed greater consonant timing abnormalities (VOT: *p* = 0.03) compared to controls. No other significant differences between groups were found for speech assessment.

In the single‐task gait assessment, PIGD subtype manifested shorter stride length (*p* = 0.03) compared to controls (Figure 1B). In the dual task, PIGD subtype had slower gait velocity (*p* < 0.001), decreased cadence (*p* = 0.01), and shorter stride length (*p* = 0.01) compared to controls. Also, TD subtype showed slower gait velocity (*p* = 0.03) and shorter stride length (*p* = 0.03) compared to controls. No significant differences between PIGD and TD subtypes for gait assessment were detected.

### 3.3 Relationships between speech and gait

**FIGURE 2:**
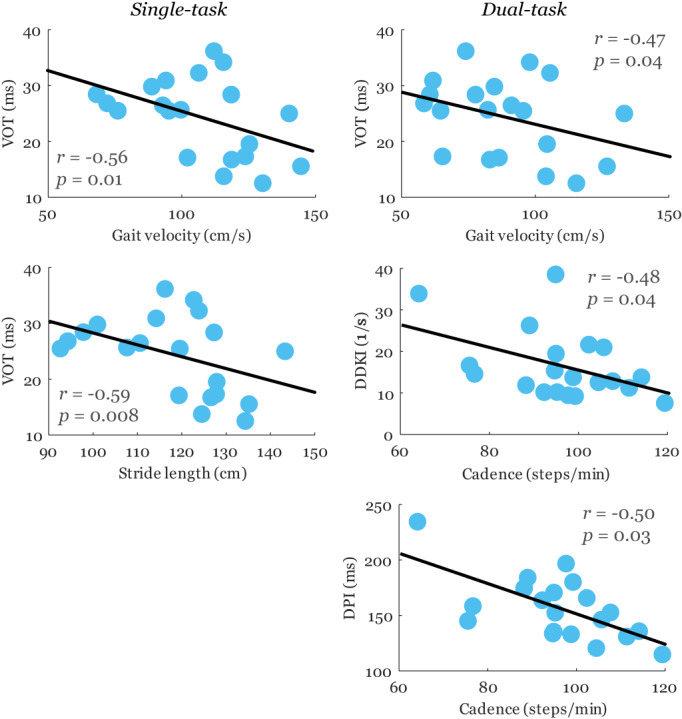
Significant correlations between speech and gait measures in the PIGD subtype. The blue circles demonstrate the real, uncorrected gait and speech values while the correlation coefficient *r* and its corresponding *p*‐value are corrected to age. DDKI, diadochokinetic irregularity; DPI, duration of pause intervals; VOT, voice onset time.

Significant correlations between speech and gait abnormalities corrected for age were found only in the PIGD subtype (Figure 2), while no relation between patterns of speech and gait disorders was observed in the TD subtype (Table 3). Specifically, consonant timing abnormalities (VOT) were related to slower gait velocity (*r* = −0.56, *p* = 0.01) and shorter stride length (*r* = −0.59, *p* = 0.008) during single task and to slower gait velocity (*r* = −0.47, *p* = 0.04) during dual task. Greater AMR irregularity (DDKI) was associated with decreased cadence (*r* = −0.48, *p* = 0.04) during dual task. Finally, prolonged pauses (DPI) were correlated to decreased cadence (*r* = −0.50, *p* = 0.03) in dual task. The same relationships but with considerably lower correlation power with r of up to −0.36 between consonant timing abnormalities (VOT) and gait parameters were also found for the entire PD group (Table 3).

**TABLE 3:** Correlations between speech and gait measures in the TD and PIGD subtypes and entire PD group.

Deviant speech dimension (acoustic feature)	Single‐task			Dual task		
	Gait velocity	Cadence	Stride length	Gait velocity	Cadence	Stride length
TD (*n* = 40)						
Pace acceleration (RA)	0.06 (0.72)	0.17 (0.31)	−0.08 (0.63)	0.13 (0.44)	0.14 (0.41)	0.11 (0.50)
Pace instability (RI)	−0.17 (0.31)	−0.09 (0.60)	−0.07 (0.66)	−0.19 (0.24)	−0.25 (0.12)	−0.17 (0.31)
Slow alternating motion rates (DDKR)	0.02 (0.91)	−0.07 (0.69)	0.13 (0.44)	0.16 (0.34)	0.04 (0.81)	0.16 (0.34)
Irregular alternating motion rates (DDKI)	−0.11 (0.50)	0.03 (0.84)	−0.19 (0.24)	−0.19 (0.24)	−0.19 (0.24)	−0.16 (0.32)
Imprecise consonants (VOT)	−0.12 (0.46)	−0.13 (0.42)	0 (0.99)	−0.16 (0.34)	−0.14 (0.40)	−0.10 (0.54)
Prolonged pauses (DPI)	−0.07 (0.67)	−0.02 (9.92)	−0.02 (0.90)	−0.10 (0.54)	−0.01 (0.94)	−0.13 (0.41)
Abnormal articulation rate (NSR)	0.18 (0.29)	0.17 (0.30)	0.08 (0.63)	0.23 (0.16)	0.23 (0.15)	0.16 (0.33)
PIGD (*n* = 20)						
Pace acceleration (RA)	−0.13 (0.62)	0.05 (0.85)	−0.22 (0.37)	−0.37 (0.12)	−0.27 (0.26)	−0.33 (0.16)
Pace instability (RI)	−0.24 (0.33)	−0.12 (0.62)	−0.21 (0.39)	−0.11 (0.65)	0.16 (0.53)	−0.27 (0.26)
Slow alternating motion rates (DDKR)	0.02 (0.94)	0.03 (0.90)	0.06 (0.80)	0.12 (0.64)	0.36 (0.13)	−0.08 (0.74)
Irregular alternating motion rates (DDKI)	−0.39 (0.10)	−0.33 (0.17)	−0.34 (0.16)	−0.41 (0.08)	**−0.48 (0.04)**	−0.22 (0.37)
Imprecise consonants (VOT)	**−0.56 (0.01)**	−0.38 (0.11)	**−0.59 (0.008)**	**−0.47 (0.04)**	−0.33 (0.17)	−0.29 (0.22)
Prolonged pauses (DPI)	−0.20 (0.41)	−0.18 (0.46)	−0.21 (0.40)	−0.42 (0.08)	**−0.50 (0.03)**	−0.06 (0.82)
Abnormal articulation rate (NSR)	−0.04 (0.87)	−0.17 (0.48)	0.04 (0.84)	−0.12 (0.64)	0.04 (0.86)	−0.25 (0.31)
All PD (*n* = 60)						
Pace acceleration (RA)	0.02 (0.86)	0.17 (0.20)	−0.10 (0.46))	0 (0.99)	0.07 (0.62)	−0.05 (0.72)
Pace instability (RI)	−0.17 (0.21)	−0.09 (0.50)	−0.10 (0.45)	−0.18 (0.16)	−0.15 (0.27)	−0.19 (0.15)
Slow alternating motion rates (DDKR)	0.04 (0.73)	−0.02 (0.87)	0.09 (0.51)	0.06 (0.65)	0.05 (0.72)	0.08 (0.55)
Irregular alternating motion rates (DDKI)	−0.26 (0.05)	−0.12 (0.38)	−0.26 (0.05)	−0.25 (0.05)	−0.23 (0.08)	−0.19 (0.14)
Imprecise consonants (VOT)	**−0.36 (0.005)**	−0.25 (0.06)	**−0.28 (0.03)**	**−0.29 (0.03)**	−0.18 (0.17)	−0.22 (0.09)
Prolonged pauses (DPI)	−0.13 (0.34)	−0.07 (0.58)	−0.11 (0.40)	−0.22 (0.09)	−0.15 (0.25)	−0.13 (0.34)
Abnormal articulation rate (NSR)	0.05 (0.73)	0.02 (0.87)	0.01 (0.91)	0.07 (0.58)	0.11 (0.41)	−0.02 (0.87)

*Note*: Data are represented by correlation coefficient *r* (*p* value). Bold numbers indicate significant correlations after adjustment by age.

Abbreviations: DDKI, diadochokinetic instability; DDKR, diadochokinetic rate; DPI, duration of pause intervals; NSR, net speech rate; PD, Parkinson's disease; PIGD, postural instability/gait difficulty subtype of Parkinson's disease; RA, rhythm acceleration; RI, rhythm instability; TD, tremor dominant subtype of Parkinson's disease; VOT, voice onset time.

## 4 DISCUSSION

In this quantitative, objective instrumental study, we attempted to detect the associations that may reflect distinctive pathophysiological mechanisms of speech and gait abnormalities in different motor subtypes of de‐novo PD. In general, our findings support the assumption that objective spatiotemporal measures of gait are more affected in the PIGD subtype compared to the TD subtype. However, in agreement with previous research,^26^ this separation based on objective assessment is not fully consistent with traditional clinical classification scheme as there were large overlaps in multiple gait parameters between PIGD and TD subtypes. Considering speech assessment, current results are principally in agreement with previous research demonstrating that patients with the PIGD subtype manifest decreased voice quality, articulatory decay, and more timing abnormalities, apart from monopitch and irregular AMR presenting in the TD subtype as well.^16^ Despite the effect of the TD/PIGD phenotype was not perceptually identifiable using the MDS‐UPDRS III speech item, the objective acoustic evaluation revealed that the PIGD subtype generally manifested more severe temporal speech impairment compared to the TD subtype. Likely because of more advanced and wide‐ranging gait and speech disorder, the significant association between gait and speech temporal impairment across several objective measures was detected only in the PIGD subtype. The relevance of this finding can be further supported by the fact that the same strength of correlations between gait and speech parameters was not observed across the entire PD group, despite the lack of significant differences between TD and PIGD subtypes in some speech and gait variables.

Namely, in the PIGD subtype, the relationships were detected between single‐task gait velocity and stride length and consonant timing abnormalities. Voicing onset time represents a temporal measure of coordination of speech articulation and voicing and is defined as the time interval between the initial articulatory release of a stop consonant and the onset of vocal fold vibration.^27^ In a similar principle as hypokinesia in PD underlies slower gait speed and shorter strides, slowing of lip and tongue movements results in a longer time required to pronounce individual consonants. This is especially evident when PD patients are challenged by the fast syllable repetition paradigm, which is designed to measure the motor abilities of speech articulators and reveal their movement limitations.^22^

Furthermore, in the dual task in PIGD subtype, additional relationships were found between decreased cadence of gait and prolonged pauses as well as decreased diadochokinetic regularity. These associations likely reflect rhythmic disorder that has been repeatedly reported in both gait and speech.^11^, ^28^ Similarly to the decrease of the number of steps, the PIGD patients slowed the speech tempo by increasing the length of pauses during reading and provided more variable pause gaps between following vocalizations during rapid syllable repetition. Indeed, dual‐ task involving serial sevens subtraction likely well represents motor‐cognitive links, similarly as the production of pauses during connected speech reflects both speech‐motor execution and cognitive‐linguistic processing. However, the deficits in pauses and diadochokinetic regularity in PIGD were not significantly different from the controls. Yet, these observed associations might still be explained by the fact that the dual‐task walking was more cognitively challenging, as cadence in PIGD was significantly altered from controls only during the dual‐task paradigm. Although we did not detect cognitive differences between PIGD and TD subtypes in our de‐novo PD cohort, it is well known that PIGD subtype poses higher risk of cognitive decline over time.^29^ Therefore, lower cadence in the dual task observed in our PIGD subtype might be interpreted as a subliminal precursor of future cognitive decline.

It remains to be elucidated why the observed speech and gait associations occurred in the PIGD subtype but not in the TD subtype. Patients with PIGD tend to exhibit a more rapid decline in motor and cognitive function compared to TD, which is considered a less severe subtype with a better prognosis.^30^ Neuroanatomically, distributed regional gray matter atrophy has been demonstrated in the PIGD, with the disruption in the precentral gyrus hypothesized to be involved in postural control, balance, and gait.^31^ In this context, it is noteworthy that acquired motor speech impairment has traditionally been associated with damage to contiguous regions, namely the left inferior precentral cortex, as corroborated by modern imaging studies.^32^ Speech disorder thus might be attributed to different pathophysiological mechanisms underlying the appendicular (limb tremor, bradykinesia, and rigidity) and axial motor symptoms of PD. In particular, temporal speech deviations such as prolonged voicing onset time seem to represent the axial speech symptom as it does not respond to dopaminergic medication and thus cannot be solely attributable to more advanced nigrostriatal degeneration.^13^ Therefore, as in the case of gait dysfunction, more widespread brain damage affecting extranigral cortical and/or subcortical regions appears to contribute to temporal speech deviations. We may thus hypothesize that more advanced alpha‐synuclein pathology spreading into cortical areas, consistent with Braak stage 4 or higher, contributes to shared pathomechanisms underlying temporal speech and gait dysfunction.

Previous findings have been mostly limited to observed correlations between freezing of gait and freezing of speech (i.e., stuttering‐like speech dysfluencies) or oral festination and festination of gait in later PD stages.^7^, ^8^, ^9^, ^10^ The present early‐stage PD cohort was free of such abnormalities of gait and speech during clinical examination, and particularly did not show any tendency toward speech acceleration as measured via the rhythmic speech paradigm. This agrees with previous studies reporting low occurrence of oral festination events in early‐stage PD,^33^ as well as the correlation of the severity of pace acceleration to motor deficits and disease duration.^24^, ^28^ Accordingly, it has been shown that PD patients exhibited significantly more dysfluent speech events compared to controls after an average time of 4.5 years of dopaminergic therapy after diagnosis.^34^

Oral festination and short “rushes” of speech in PD are closely connected with changes in speaking rate.^35^ Our observation of increased speech rate across both fast syllable repetition and reading in the PIGD compared to TD subtype might provide a further piece of the puzzle regarding the discrepant findings about speaking rate in PD in the literature.^36^ This finding appears to be well following differences between PD motor subtypes detectable on neuroimaging. Structural connectivity analysis has shown that patients with PIGD have alterations of cortico–basal ganglia pathways while TD do not.^37^ Therefore, more cortical brain atrophy is likely responsible for the faster speech rate observed in the PIGD subtype, which might be a precursor of events such as oral festination that may develop later in the disease process. On the other hand, TD patients show greater cerebello‐thalamo‐cortical circuitry dysfunction.^38^, ^39^ Previous study has demonstrated that the extent of cerebellar atrophy is related to a reduced rate,^40^ likely due to a reduced ability to program movement sequences in advance of movement onset.^41^ These assumptions can be further supported by neuroimaging finding that the various cortical and subcortical brain regions engaged in speech motor control might be organized into two separate networks (medial and dorsolateral premotor cortex, anterior insula, and superior cerebellum versus sensorimotor cortex, basal ganglia, and inferior cerebellum), most presumably responsible for motor preparation and execution processes.^42^ Since both loops are involved in the rate of the produced syllable trains and associated rhythmic modulations,^42^ they might also help to explain clinical observations of accelerated or even slowed speaking rate in PD. However, it is challenging to differentiate the problems from rhythm generation (internal cueing of rhythm) and the initiation/bradykinesia issues that arise from external/motoric factors.

A limitation of this single‐center study is that our sample comprises only 20 patients with PIGD, as this subtype is relatively rare in de‐novo PD. This is in accordance with the PPMI cohort consisting of a large sample of de‐novo PD, where TD represents the dominant subtype compared to PIGD.^43^ Thus, future research based on a greater sample, preferably obtained via multicenter cohorts, should confirm and further elaborate our findings. It would also be preferable to support the present findings by neuroimaging data to substantiate the cortical involvement in gait/speech rhythm disorder.

In conclusion, this study underlines several common features of gait and speech abnormalities in PD specific to the PIGD subtype which are detectable already in the early disease stage using quantitative objective measures. Our findings suggest a similar physiopathological process in the temporal coordination of speech articulation and voicing and gait velocity as well as in speech and gait rhythm disorder. Future longitudinal studies should investigate whether speech patterns might provide early pathophysiological hallmarks and biomarkers of PD motor subtypes and predict the possible conversion into PIGD subtype.^39^

## AUTHOR CONTRIBUTIONS

Research project: (A) Conception: J.R., and E.R. (B) Organization: J.R., P.D., and E.R. (C) Execution: J.R., R.K., S.V., T.T., M.N., J.N., and P.D. Statistical analysis: (A) Design: J.R. (B) Execution: J.R. (C) Review and critique: R.K., S.V., T.T., M.N., J.N., P.D., and E.R. Manuscript: (A) Writing of the first draft: J.R. (B) Review and critique: R.K., S.V., T.T., M.N., J.N., P.D., and E.R.

## CONFLICT OF INTEREST STATEMENT

All authors report no conflict of Interest concerning the research related to the manuscript.

## Supporting information


Video S1.

## Data Availability

Individual participant data that underlie the findings of this study are available upon request to the corresponding author by qualified researchers (i.e., affiliated to a respected university or research institution/hospital). The speech data are not publicly available due to their contain of information that could compromise the privacy of study participants.
